# Varied Probability of Staying Collapsed/Extended at the Conformational Equilibrium of Monomeric Aβ_40_ and Aβ_42_

**DOI:** 10.1038/srep11024

**Published:** 2015-06-05

**Authors:** Wanling Song, Yuanyuan Wang, Jacques-Philippe Colletier, Huaiyu Yang, Yechun Xu

**Affiliations:** 1 CAS Key Laboratory of Receptor Research, Drug Discovery and Design Center, Shanghai Institute of Materia Medica, Chinese Academy of Sciences (CAS), Shanghai 201203, China; 2 Univ. Grenoble Alpes, IBS, F-38044 Grenoble, France; 3 CNRS, IBS, F-38044 Grenoble, France; 4 CEA, IBS, F-38044 Grenoble, France

## Abstract

In present study, we set out to investigate the conformation dynamics of Aβ_40_ and Aβ_42_ through exploring the impact of intra-molecular interactions on conformation dynamics using equilibrium molecular dynamics simulations. Our 40 microsecond-scale simulations reveal heterogeneous conformation ensembles of Aβ_40_ and Aβ_42_ that encompass ~35% β-strand and ~60% unstructured coils. Two conformational states were identified in both alloforms: a collapsed state (CS) that resembles the structural motif of face-to-face hydrophobic clustering in amyloid fibrils, and an extended state (ES) that features the structural characteristics of anti-parallel β-sheets in amyloid oligomers. In Aβ_40_, the C-terminus remains unstructured and rarely interacts with other parts, thereof the hydrophobic clustering is in loose contact and the peptide assumes ES with high probability. In contrast, the C-terminus of Aβ_42_ adopts a β-strand structure that strongly interacts with segments E3-R5 and V18-A21. The active association leads to a more compact hydrophobic collapse and refrain the alloform from ES. Based on the structural characterization, we propose that the fibril and oligomer assembly pathways could respectively take off from CS and ES, and their aggregation propensity may be governed by the probability of visiting the corresponding conformational states at the equilibrium.

Alzheimer’s disease (AD) is the most frequent cause of senile dementia. The two histological hallmarks of the disease are the appearance of extracellular senile plaques and intracellular neurofibrillary tangles in victims’ brains^1^,^2^,^3^. The principal constituent of the plaques is the β-amyloid peptides (Aβs) produced by sequential action of β- and γ-secretases on the amyloid precursor protein (APP)^4^,^5^. Aβ_40_ and Aβ_42_ are the two most abundant Aβ isoforms in the plaques, with the only difference found at the C-terminus where the latter has two more hydrophobic residues (I41 and A42). It was demonstrated that Aβ_42_ is more neurotoxic than Aβ_40_^6^,^7^,^8^ and an increased Aβ_42_/Aβ_40_ concentration ratio correlates with the onset of AD^9^. The increased neurotoxicity of Aβ_42_ has been proposed to stem from its significantly higher aggregation propensity^10^,^11^,^12^. The molecular basis for this phenomenon remains unclear but likely originates from differences in the conformation dynamics of the two peptides.

Aβ_40_ and Aβ_42_ form β-sheet-containing structures that assemble into a variety of polymorphic oligomers and fibres. Solid-state NMR showed that they both adopt similar U-bent β-sheet rich structures in protofilaments, wherein side chains of residues of the central hydrophobic cluster (CHC, L17-A21) contact those of the C-terminal hydrophobic region (A30-A42)^13^,^14^. Apart from sharing a U-turn at E22-K28, specific intra- and inter-molecular interactions, however, differ within and between the Aβ_40_ and Aβ_42_ protofilaments^13^,^14^,^15^,^16^. X-ray microcrystallography provided atomic-resolution insights into the structures of Aβ fibre-prone segments, uncovering the molecular basis for Aβ fibre polymorphism^17^. By combining atomic structures with previous nuclear magnetic resonance^13^,^17^,^18^ and cryo-EM studies^16^, several models were constructed for Aβ fibres^19^,^20^.

In contrast to the well-resolved protofilaments, our understanding to structures of the monomeric Aβ_40_ and Aβ_42_ is limited. High-resolution data under physiological conditions are absent, due to the pronounced aggregation propensity and hydrophobicity of Aβ. Instead, much information, regarding the secondary structures, long-range contacts and flexibilities, are gathered by NMR based on various Aβ fragments with improved solubility or in more hydrophobic conditions^12^,^21^,^22^,^23^,^24^,^25^,^26^,^27^. For example, in water, Aβ_21–30_ takes a bended structure with a β-turn at V24-L28^28^; Aβ_10–35_ folds into a collapsed coil where Y10-H14 and V24-N27 condense around the CHC region (L17-A21) in extended structures^24^. In aqueous sodium-dodecyl sulphate (SDS) micelles, which is supposed to simulate the water-membrane environment after γ-secretase intra-membrane action, residues Q15-V36 of Aβ_40_ take on a helical conformation with a hinge a residues G25-N27, while D1-H14 adopt unstructured, extended conformations^29^.

Characterizing the conformation dynamics of monomeric Aβ_40_ and Aβ_42_ is a pre-requisite to comprehending the Aβ self-assemble pathway and the differences between the two peptides. The demand on the structural information of full-length Aβ has encouraged the application of molecular dynamics (MD) simulations. Enhanced sampling methods were developed to explore the highly diverse conformational ensembles accessible to Aβs^30^,^31^,^32^. Several noteworthy studies include that Vitalis *et al.* conducted multiple replica-exchange Monte Carlo simulations with implicit solvent model, from which a micelle-like architecture of Aβs monomers was revealed^33^; Sgourakis *et al.* and Ball *et al.* respectively performed replica-exchange molecular dynamics simulations in conjunction with NMR data and confirmed the existence of structured region in these two intrinsically disordered peptides^30^,^34^,^35^; more recently, Lin *et al.* deployed Markov state model analysis based on extensive *in silico* samplings and observed reduced β-hairpin formation near C-terminus of the shorter Aβ monomer^36^. Admittedly, the structures of Aβs monomers have been probed by several computational studies and a further one of such may seem difficult to justify at first sight. However, the structural differences between Aβ_40_ and Aβ_42_ revealed so far appear surprisingly trivia in comparison to the apparent gaps in their aggregation propensity. In fact, most *in silico* studies that succeeded in discriminating the two alloforms remained equivocal in interpreting the structural information in terms of understanding Aβ aggregation. This predicament comes from two points: i/ the force field bias and ii/ the complexity of Aβ conformational space. While the former contributes to significant lower content of β-sheet (~6%–7%) in comparison to the estimation from CD experiments (~30%)^37^ and may lead to biased samplings^38^, the latter requires such large volumes of samplings for a generalized understanding that may go beyond our current computation limits. Thus, characterizing the conformation dynamics and understanding the structural differences of Aβs call for alternative ways.

In current study, we set out to investigate the impact of intra-molecular interactions on the dynamics of Aβs using equilibrium MD simulations and an atomistic force field from Gromos family (Gromos 53a6 ff). We implemented 40 microsecond-scale molecular dynamics simulations on Aβ_40_ and Aβ_42_ (70.6 μs in aggregate simulation time), allowing unprecedented insights into their equilibrium dynamics. The simulation data showed improved consistency with three-bond J-coupling constants and residual dipolar couplings than previous works. The secondary structure profiles from our simulations that comprise of ~35% in β-strand and ~60% as random coils are also in better agreement with CD estimations. Spectral clustering revealed that both isoforms exists in two states: i/ a collapsed state (CS) held together through hydrophobic clustering; and ii/ an extended state (ES) populated with higher orders of β-strands. The C-terminal β-hairpin of Aβ_42_ diminishes the overall conformational entropy via stabilizing the peptide in the collapsed state. In contrast, the C-terminus of Aβ_40_ only shows weak intra-molecular interactions. The shorter peptide, hence, enjoys more efficient transition to the extended state. The resemblance of CS to amyloid fibril and ES to the observed oligomers indicates that they respectively could be the starting point of fibril and oligomer assembly pathways. Our results thus suggest that the aggregation propensity of amyloid peptides may be encoded in the probability of adopting the corresponding conformational states at the equilibrium.

## Results

### Validation and characterization of the conformation ensembles

Two sets of simulations were generated for both Aβ_40_ and Aβ_42_, each containing ten trajectories (Table 1). In the first set of simulations (Group A and C for Aβ_40_ and Aβ_42_, respectively), the starting models were taken from the coil structure of Aβ_10–35_ in water with necessary residues added manually^24^. The second set of simulations (Group B and D for Aβ_40_ and Aβ_42_, respectively) was based on the helical structure of Aβ_40_ in SDS micelles^29^. Using two NMR structures of monomeric Aβ allows diversifying our conformational sampling while reducing the starting model bias during the simulations. Groups from the same structural set were extended to similar lengths, while to account for the time required for helix unreeling, the helix-starting set were systematically run longer than the other. In the first 200 ns of simulations, large structural changes occur in both Aβ_40_ and Aβ_42_ (Fig. S1), thus corresponding data were discarded. Using a conformation-saving interval of 100 ps, the resulting number of sampling points are 154,481, 176,112, 154,392 and 187,712 in Group A, B, C and D, respectively.

**Figure 1:**
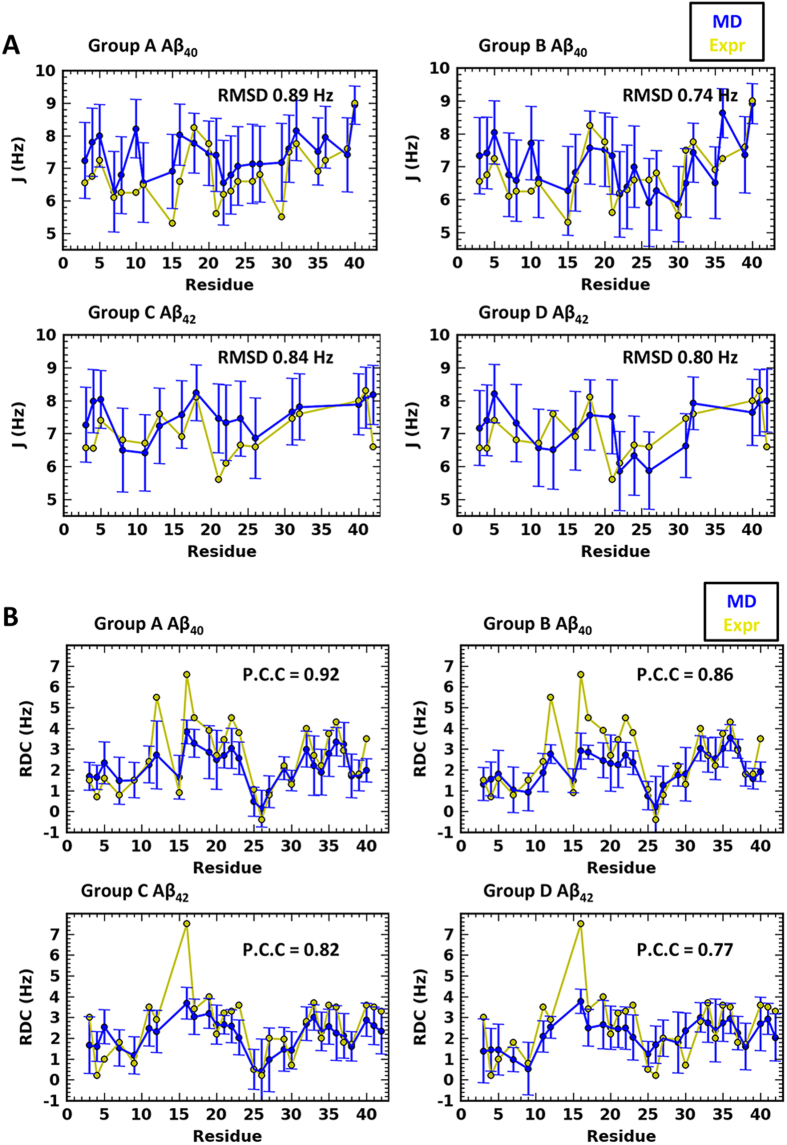
Validation with NMR measurements. The back-calculated J-coupling values (**A**) and residual dipole coupling constants (**B**) from our simulation coordinates are compared with the NMR data from Yan *et al.*^39^. Simulations data are shown in blue circles and experimental data yellow ones. The error bars denote the standard deviations from the 10 trajectories in each simulation group.

Validation of the samplings was made through comparisons of the back-calculated three-bond J-coupling constant 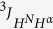 and residual dipole couplings (RDCs) with the experimental measurements from Yan *et al.*^39^. We examined different parameter sets for the Karplus equation in calculating J-coupling as described in Method. In general, good consistency was observed between the experimental and back-calculated data. The RMSD in the best-fitting set (Fig. 1A) is ~0.78 Hz for Aβ_40_ and ~0.82 Hz for Aβ_42_. The result is lower than or comparable to those using other force fields^30^,^34^,^35^. Residues Q15, A21 and A30 in Aβ_40_, and A21 in Aβ_42_ exhibit the largest deviation. These residues locate at two different sets of complex environment: Q15 at a very flexible region with multiple charges and A21/A30 at hydrophobic patches with charged residues at vicinity. That these outliners are also observed in previous simulations may reflect some common limitations among modern force fields in taking care of the interplay of multiple factors. Residual dipole coupling constants were calculated by PALES^40^ using global alignment. The RDCs are in excellent agreement with the experimental data, showing an average RMSD of ~1.06 Hz for Aβ_40_ and ~1.16 Hz for Aβ_42_ (Fig. 1B). The Pearson’s correlation coefficient (P.C.C) reaches above 0.75 for all groups, which is a significant improvement from previous simulations. Residues on flexible loops, including V12, K16 and L17 in Aβ_40_ and Q15 in Aβ_42_, exhibit the largest deviation. The discrepancy may result from the intrinsic uncertainty of backbone orientation of the flexible loop. Taken together, the good agreement with the NMR measurements indicates that our simulations have sampled the correct conformational shapes of Aβ_40_ and Aβ_42_.

**Figure 2:**
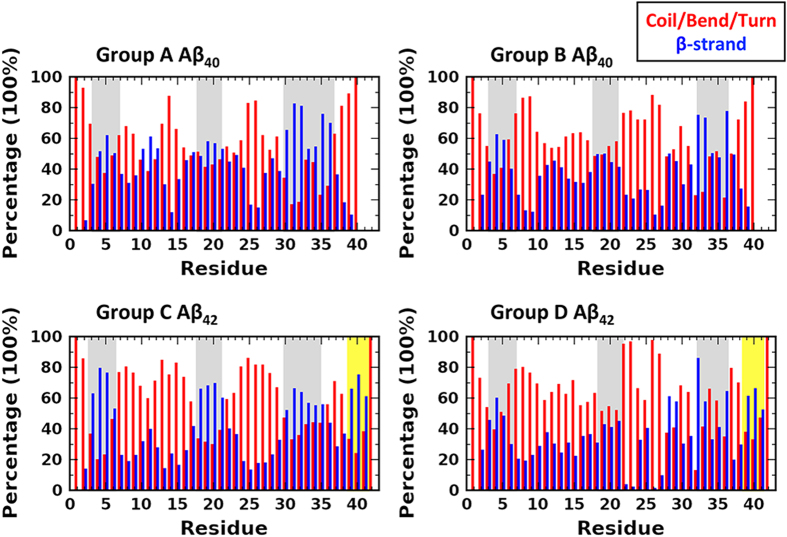
Secondary structure occupancy by residue. Red and blue bins are the percentage of structural elements of coil/bend/turns and β-strand occupying each residue, respectively. The β-strand regions shared by four groups are highlighted in gray, and the extra β-strand in Aβ_42_ is in yellow.

The secondary structure occupancy by residue was calculated using DSSP^41^. In accordance with the solution NMR analyses, the difference between the two alloforms is subtle. The two peptides share three regions with the highest propensity of forming β-strands, locating at E3-R5, L17-A21 and I31-M35, and in-between regions of turns and bends centring at D7-G9, H13-Q15 and V24-N27 (Fig. 2). The charged residues at the turns and bends form several most stable hydrogen bonds in both peptides, including D7 with S8, H14 with K16, and E22/D23 with S26/N27-K28, while other steady hydrogen bonds are observed between adjacent contacts, e.g. E3 with R5, and E11 with H13 (Fig. S2 and S3). The most striking difference in the secondary structures of the two peptides lies at the C-terminus, in agreement with NMR findings^21^. In Aβ_42_, residues V39-A42 folds into an additional, extremely resilient β-strand, whereas its counterpart in the shorter alloform (G37-V40) remains largely unstructured.

The content of secondary structure elements was also calculated. According to the group-averaged profiles, our samplings of Aβ_40_ and Aβ_42_ are dominated by two sets of structural elements: coil/bend/turn and β-strands, whose occupancies in these two alloforms are similar (Fig. S4 and Table S1). In the coil-starting groups (A and C), the occupancy of coil/bend/turn and β-strand are ~58% and ~41% respectively, while in the helix-starting groups (B and D), they are ~62% and ~36% respectively. The helical content is low, remaining at ~1%, whereas a slight increase of the content is observed in the helix-starting groups, due to the unreeling process. Notably, the secondary structure profiles display large discrepancy with those from previous simulations on Aβs that comprised of ~6–7% β-sheet and ~20–30% helix^30^,^34^. But the data from the present simulations are indeed in better consistency with the CD spectroscopy results that reveal the two alloforms containing ~30% β-strand and 5% helix^37^,^42^. Thereof, our simulations, in conjunction with the experiments, suggest that higher content of β-strand presents in Aβ monomers than the calculation from previous simulations, and accordingly raise questions about the hypothesis that the rate-limiting step of amyloid aggregation lies in the transition to conformations with higher β-strand-content at monomer level.

### Two conformational states in Aβ_40_ and Aβ_42_

A spectral clustering approach designed by Sgourakis *et al.*^35^ were used to characterize the conformational states in each group. This clustering approach is based on pair-wise contact information rather than the calculation of RMSD, and is advised in the studies on small intrinsically disordered peptides like Aβs, which experience drastic changes in molecular shapes during simulations and depower clustering strategies based on geometry distance. Following the procedures introduced in the paper, we obtained a small number of eigenvectors that can be used to cluster the conformations into groups that share interaction patterns. A direct visualization of the conformations in the space defined by the three most discriminative eigenvectors is shown in Fig. S5. Each group of conformation ensembles were clustered into ten interaction patterns (See Methods for the discussion on the cluster numbers), and the conformations at the centroid of all clusters are reported in Fig. S6-S9.

**Figure 3:**
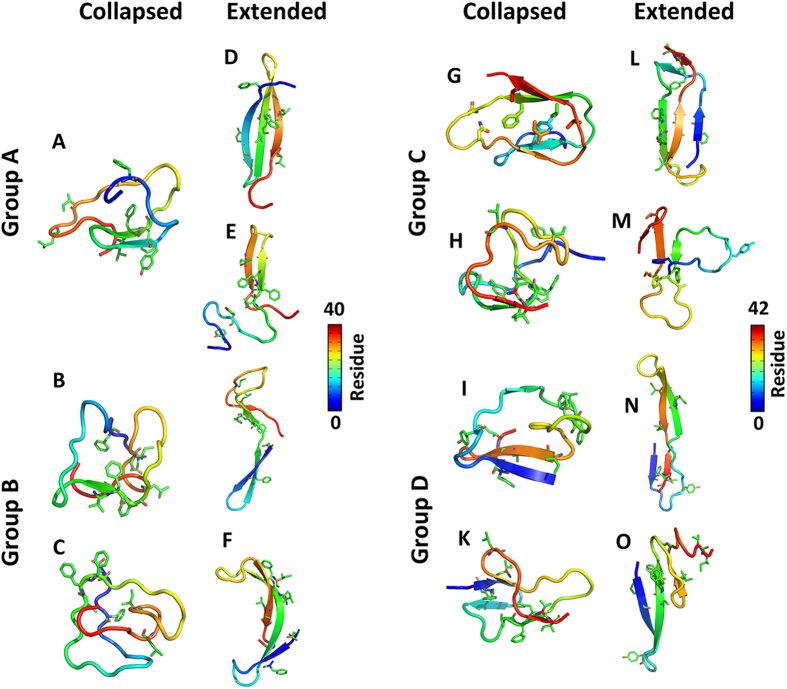
The typical collapsed and extended conformations in Aβ_40_ and Aβ_42_. Peptides are colored in rainbow based on Cα atoms. The hydrophobic residues that are frequently observed in the hydrophobic clustering, including F4, Y10, V12, F19, F20, A21, I31, L34, and V36, are shown in sticks.

The 40 representative conformations showed high heterogeneity in loop and β-sheet arrangements, recapitulating conformational heterogeneity from experiments. We observed two dominant conformational states in Aβ_40_ and Aβ_42_: a collapsed state (CS) and an extended state (ES), although the specific residue-residue associations differ among clusters from the two alloforms. A couple of typical conformations in each state are shown in Fig. 3. In the CS, hydrophobic residues, most frequently including F4, V18, F20, A21, I31, L34 and V36, collapse into a core that forms the base of a highly dynamic globule at the surface of which polar and charged residues stand facing the solvent. The collapse contributes a main stabilizing factor to the state, while also gives rise to the dynamics, *i.e.* the disordered state, of the conformations. A variety of residue associations are observed in both alloforms. In Aβ_40_, the hydrophobic collapse can involve β-strands V18-A21/A30-L34 and loop F4-D7 (Fig. 3B), or β-strands Y10-H13/V18-A21 and loops F4-D7/G29-I32/M35-G37 (Fig. 3A). In Aβ_42_, the participants can be grouped into two sets: one involving two β-sheets formed by G38-I41/V18-A21, and A30-G33/G9-V12 (Fig. 3G,H), while the other two β-sheets by V39-I41/E3-R5, and L17-A21/K28-L34 (Fig. 3I). Apart from the hydrophobic stacking, polar contacts also play an important role in maintaining the conformations. The conserved interactions of H6-S8 and V24-K28 stabilize the required turns/loops that bring distal hydrophobic patches in vicinity.

**Figure 4:**
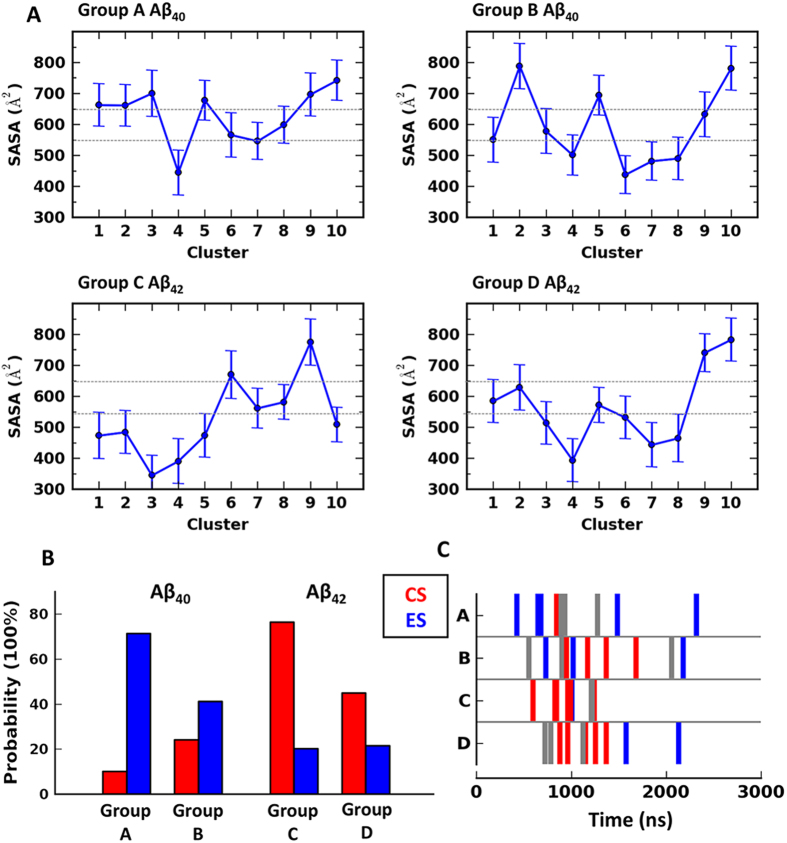
Varied distribution of collapsed/extended states in Aβ_40_ and Aβ_42_. **A**) The solvent accessible surface area (SASA) of the residues that are most frequently observed in the hydrophobic collapse of both alloforms, *i.e.* F4, V18, F20, A21, I31, L34 and V36. The SASA is averaged from all the snapshots in each cluster. The error bars denote standard deviation within the cluster. **B**) The probability of observing collapsed/extended states in each group. The collapsed state is defined as SASA below 550 Å^2^, and the extended state as SASA above 650 Å^2^. **C**) The average appearance time of each cluster in the course of simulations. The clusters in collapsed state are colored red and in extenddd state colored blue. Those between the two states, *i.e.* SASA between 550 Å^2^ and 650 Å^2^, are in gray.

The CS revealed by our samplings share similarities with the micelle-like conformations reported by Vitalis *et al.*^33^ in that hydrophobic residues collapse into a dynamics core. But three major differences come up under closer comparison. Firstly, higher content of β-strand is revealed in our samplings. In particular, the N-terminal regions of both Aβ_40_ and Aβ_42_ are frequently occupied by β-strand in our simulations, and more active in intra-molecular associations with other parts of the peptide. An entirely unstructured and unpaired N-terminus as proposed by Vitalis, we suggested that, is not very likely, since this region populates multiple charged residues that are capable of forming polar contact with the rest of the peptides (Fig. S2 and S3). Plus, a structured N-terminus is also supported by the ^1^H NMR data from Danielsson *et al.* that provide evidence of Aβ_40_ N-terminus taking β-strand conformation at room temperature^43^. Secondly, other than entirely collapsed coils, the CSs in our samplings prefer a roll shape in which hydrophobic residues contact the solvent at two flanks. Such an arrangement is reminiscent of the monomer structure in amyloid fibril. In particular, the two β-strands in the fibril constructs, *i.e*. V18-A21 and A30-M35, are frequently involved in the hydrophobic collapse of both alloforms. Thirdly, distinct structural differences between Aβ_40_ and Aβ_42_ are observed. In Aβ_40_, the present of loops in the hydrophobic collapse brings in flexibility and leads to loose internal structures in the conformations. In comparison, the CS of Aβ_42_ has a more structured collapse that results in compact conformations and reduced solvent exposure of hydrophobic residues. As shown by the solvent accessible surface area (SASA) of the hydrophobic residues (Fig. 4A), typical CS conformations are observed below 550 Å^2^ in both alloforms. But the conformations in Aβ_42_ on average exhibit significant lower SASA than those in the shorter alloform.

**Figure 5:**
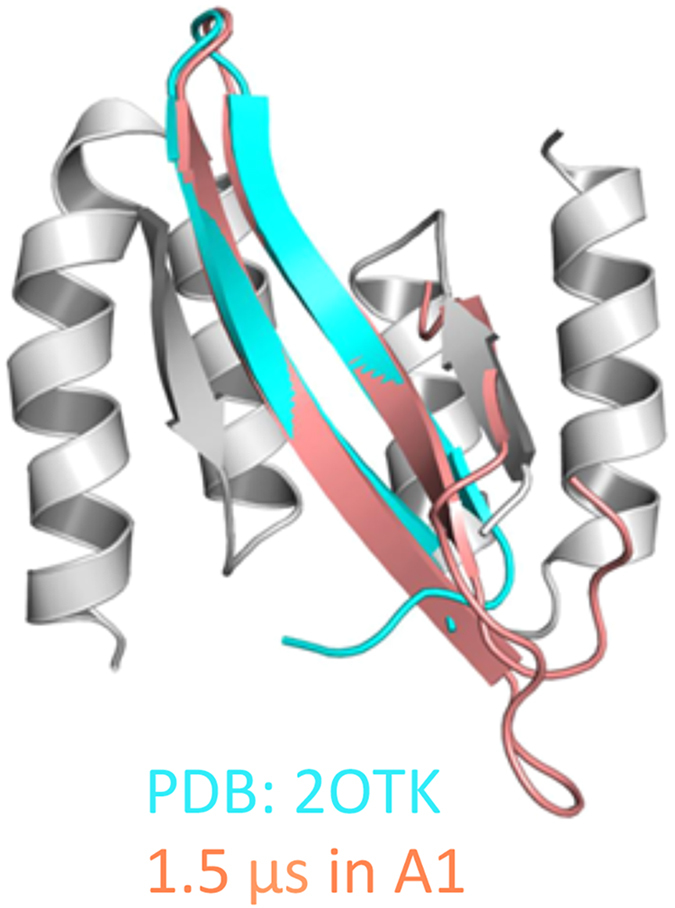
Superposition of the best-fitting ES conformation (salmon) to the NMR structure of Aβ_40_ in complex with an affibody (PDB code 2OTK) (cyan). The best-fitting conformation is from Cluster 9 of Group A. The affibody in the NMR structure is colored gray.

In the ES, the hydrophobic core breaks down, and Aβ adopts more flattened shapes where the main interactions are replaced by hydrogen bonds between β-strands. ES containing two to six antiparallel β-strands are observed, several of which reproduce the characteristics of experimentally determined Aβ oligomer structures. Notably, Cluster 9 of Group A (Aβ_40_) that comprises of two anti-parallel β-strands at V18-E22 and A30-M35 (Fig. 3E) show high similarity to the β-hairpin of Aβ_40_ stabilized by an affibody (PDB code 2OTK)^44^. The Cα RMSD between the NMR structure and the best-fitting from the cluster is 0.4 Å (Fig. 5). Apart from the NMR determined part, conformations in this cluster also have a short N-terminal hairpin (E3-H6 and Y10-V12) that is connected to the other end through a loop at H13-L17. The flexibility of H13-L17 may correspond to the unobservable NOEs and the NMR chemical shifts that are close to random coil values in the N-terminal region^44^. Cluster 6 of Group C (Aβ_42_) matches the β-meander structure proposed for an Aβ_42_ pentamer oligomer by Ahmed *et al.*^45^. In agreement with the solid-state NMR results, conformations in this cluster compose of three anti-parallel β-strands of CHC, I31-V36 and V39-I41 connected by a turn at G37-G38 and a loop at D23-K28 (Fig. 3M). The N-terminal is in unstructured loop, which is consistent with the experimental observation that the amide hydrogen-deuterium exchange rate is high in this region^45^. Other frequent ES conformations include structures of three β-strands that are contributed by F4-S8, Q15-A21 and G29-V36 in Aβ_40_ (Fig. 3D) and residues A2-H6, V18-E22 and G37-I41 in Aβ_42_ (Fig. 3L).

### Aβ_40_ and Aβ_42_ show varied probabilities of staying CS/ES

We next set out to calculate the probability of finding CS/ES in each simulation group. Solvent accessible surface area (SASA) of hydrophobic residues was used to define the two conformational states. Typical collapsed conformations are found below SASA = 550 Å^2^ and typical extended above 650 Å^2^ (Fig. 4A). Based on this criteria, the probability of Aβ_40_ staying in CS/ES is ~10%/71% and ~24%/41% in Group A and B respectively, whereas that of Aβ_42_ is ~76%/20% and 44%/21% in Group C and D respectively (Fig. 4B). Striking difference exists between the two allforms and the longer one shows obvious preference to staying in CS. To investigate the transition between CS and ES in the two alloforms, we further calculated the appearance of the two conformational states in the course of simulations. In the coil-starting groups, the first three clusters of Aβ_40_ belong to ES conformations with an average appearance ranging from 500 ns to 800 ns, indicating that the shorter alloform is able to efficiently take extended conformations from coils. The sole CS cluster of Aβ_40_ in the group has an average appearance of ~900 ns. Aβ_42_ from the similar starting configurations, however, exhibits a very different scenario. The first five clusters in the group are in CS conformations, showing an average appearance between 600 ns and 1000 ns, whereas the mere two ES clusters lie at ~950 ns and ~1200 ns respectively (Fig. 4C). The predominance suggests that the longer alloform prefers to adopt CS from coils. In the helix-starting groups, an unreeling event first takes place that gives rises to larger proportion of conformations whose SASA is between 550-650 Å^2^ (gray bars in Fig. 4C), and postpones the CS and ES. Nevertheless, the trend is similar to that in the coil-starting groups. During the unreeling, the C-terminal β-strand of Aβ_42_ are able to frequently associate with N-terminus, and create a beneficial environment that fosters hydrophobic clustering. Thus the first CS cluster in Group D averages at ~750 ns (Cluster 3), long before the two ES clusters that lie at ~1500 ns and ~2100 ns. In contrast, the unstructured C-terminus of Aβ_40_ shows significantly decreased interaction with other parts of the peptide. This leads to that the peptide is less efficient in organizing a hydrophobic collapse and in some case directly transit into ES. Thereof, the first ES in Group B (~750 ns) advances the first CS cluster (~990 ns).

### The role of the C-terminal β-strand in Aβ_42_

**Figure 6:**
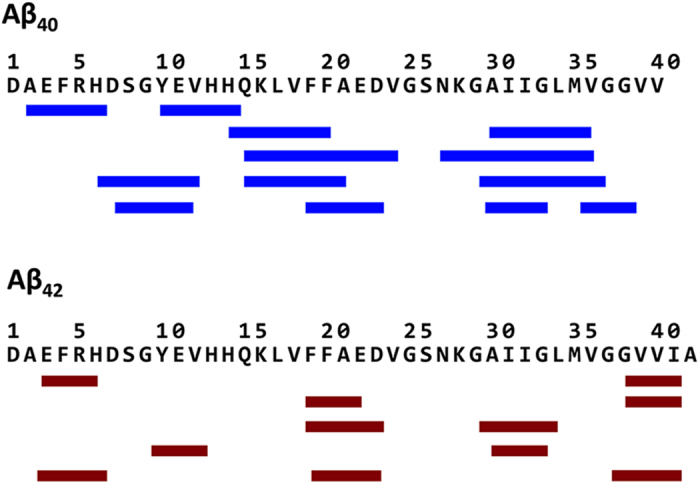
β-sheet associations. The major β-sheet associations observed in the conformation ensembles of Aβ_40_ and Aβ_42_ are labelled by strips and paired by lines.

The mere difference between Aβ_40_ and Aβ_42_, in the aspect of sequence, is the presence of two extra hydrophobic residues at the C-terminus of the latter. In line with experimental observations and previous simulations^21^,^27^,^30^,^36^,^44^, the additional two hydrophobic residues contribute to an extremely resilient β-strand in our obtained conformation ensembles of Aβ_42_ (Fig. 2). As a result, the β-strands in the rest of Aβ_42_ shift by residues in comparison to the shorter one, and the intra-molecular associations are altered. In Aβ_40_, the most frequent interactions are found between A2-H6 and Y10-H14, and between H14-F20 and A30-V36. But in Aβ_42_, as the C-terminus shows high preference to form β-sheet with E3-R5 or V18-A21, the frequent associations are instead found between V18-D23 and K28-G33 (Fig. 6). The altered associations lead to two major changes in the conformation and dynamics of Aβ_42_. Firstly, the active interaction of the C-terminal β-strand with the rest of the peptide brings C-terminal hydrophobic region and CHC region in vicinity, which creates beneficial environment that fosters the hydrophobic collapse formation. Indeed, the transition to CS is efficient in both coil- and helix-starting simulations of Aβ_42_. Plus, when the longer alloform is taking the ES, the extra β-strand also bends conformations through its intra-molecular associations. The bended shape prevents the β-strands from further elongation, and restrains the hydrophobic residues from further breaking. As a result, the transition is reversible, and the peptide is capable of returning to the CS, though less compact, after having folded into the ES (Fig. S10). Secondly, the C-terminal β-strand brings in the Aβ_42_ hydrophobic collapse higher content of β-sheet associations, the main-chain hydrogen bonds of which ‘strip seal’ the conformations. One apparent outcome of such structures is that the stability of CS is increased and the appearance of ES is delayed. In comparison, the unpaired C-terminus of Aβ_40_ significantly weakens the hydrophobic collapse, as the C-terminal hydrophobic residues are impeded from active involvement in the collapse and the resulting CS has less local structures. In addition, the conformational constrain imposed by the association of C-terminus with the rest of Aβ_42_ in ES is also relieved in Aβ_40_. The shorter alloform is able to arrange the β-strands in ES in flat tandem and easily expand into longer lengths (Fig. S10).

## Discussion

By performing 40 equilibrium MD simulations, we sufficiently expore the impact of intra-molecular interactions on conformation dynamics. The obtained conformations exhibit improved consistency with NMR measurements than previous simulations. The P.C.C between RDC values back-calculted from our coordinates and measurements from NMR reaches above 0.75, whereas previous ones show a range of 0.4–0.5^30^,^34^,^35^,^46^. The improvement is ascribed to better sampling of β-strand. We observed ~35% β-strand alongside ~60% unstructured coils, which is in good congruency with CD estimation from Fezoui *et al.*^37^. In fact, this trend has already appeared in the simulations adopting REMD method. From Sgourakis *et al.*^35^ to Rosenman *et al.*^47^, an increased agreement with NMR measurements is accompanied by samplings with more β-strands at local regions. The notion of extended structures occupying local regions also gains supports from experiments. Through two-dimensional NMR, Hou *et al.*^25^ demonstrate that the two hydrophobic regions on Aβs, *i.e.* the central hydrophobic segment and I31-V36, are occupied by β-strands. Danielsson *et al.*^43^, by using ^1^H NMR spectroscopy, provide conclusive evidence that N-terminal region and central hydrophobic segment are taking β-strand conformations at room temperature. Thus, our simulations, in conjunction with the experimental data, confirm that higher content of β-strand are indeed populated in Aβs than estimations from previous *in silico* studies.

The obtained ensembles capture a couple of distinct conformations that are reveled by several independent sutdies, including the micelle-like conformations by Vitalis *et al.*^33^ and the β-pleated structures by Hoyer *et al.*^44^ and Rosenman *et al.*^47^. We assign the former conformational state as CS and the latter as ES. As the equilibrium molecular dynamics simulations are able to sample these conformational states within one trajectory, the probability of visiting each state can be calculated. The results reveal that both Aβ_40_ and Aβ_42_ experience transitions between CS and ES. This observation is in line with the NMR readings that the two isoforms share similar *R*_*1*_, *R*_*2*_ and nuclear Overhauser enhancement (NOE) values^21^. The longer alloform shows significant higher propensity of staying in CS than the shorter one. The shifted preference results from changes in inter-molecular associations, *i.e.* more compact hydrophobic collapse, and in local conformations, *i.e.* more ordered structures in hydrophobic collapse, all of which can be unequivocally assigned back to the addition of a β-strand at Aβ_42_ C-terminus. The series of related changes brought about by the extra β-strand point out a critical role of residual interactions in shaping the conformation of Aβ, and raise doubts on the biological relevance of Aβ fragments to full-lengths. Thus extra cautions should be made when deducing the full-length structures of Aβs based on the fragments that indeed exhibit very different biophysical properties, such as the solubility and aggregation kinetics, than the former^23^,^24^,^26^,^48^.

A tormenting question in understanding amyloid peptide aggregations is why Aβ_42_, with addition of merely two residues, exhibits much pronounced aggregation propensity comparing to Aβ_40_. As plenty of experiments have demonstrated that the amyloid aggregation characterizes as an obvious nucleation-dependent process^49^, the pertinent issue in this puzzle may well become why the nucleation in the longer alloform occurs with much higher efficiency than that in the shorter one. Paravastu *et al.* showed that the molecular structure of mature fibrils is closely related to and mostly likely to reflect the structure of seedings^50^. It is thus reasonable to posit that the nucleate of amyloid fibrils should bear some of, if not all, the characteristics of mature fibril structures. The conformations in CS carry distinct face-to-face interactions of hydrophobic patches that resemble the structural motif in fibrils. Several critical interactions in fibrils are also preserved in CS, including the invariant involvement of hydrophobic patches of V18-A21 and I31-M35, and polar contact of V24-L28 at the conserved turn. Plus, from a kinetic point of view, the association of collapsed conformations is beneficial, as the merge of hydrophobic collapses could be rewarded by an entropy gain that results from reducing solvent exposure of hydrophobic residues. Based on these observations, it is tempting to propose that the nucleation takes off from CS and the structural diversity observed in CS corresponds to the differences in fibrillar architectures. As our simulation results demonstrate that the probability of Aβ_42_ staying in CS is significantly higher than Aβ_40_, the enhanced aggregation propensity of the former could result from the consequent lowered energy barrier for nucleate formation. Besides, greater resemblance to fibril structure is observed in the CS of Aβ_42_, including increased occupancy of β-strand at V18-A21 and I31-M35 and compact hydrophobic collapsed. These structural advantages could also contribute to the effectiveness of nucleation.

The ES conformations carry the signature structure of amyloid oligomers, *i.e.* antiparallel β-sheet^51^. Indeed, several clusters resemble the experimentally determined oligomer structures, including the β-hairpin in complex with an affibody by Hoyer *et al.*^44^, and the β-meandering in the disc-like pentamer by Ahmed *et al.*^45^. It was posited based on the finding of Hoyer *et al.* that the stacking of β-hairpins would transit into the structural motif of fibrils through a 90° rotation in the axis of backbone. While the intimidating energy barrier challenges such transition, experiments have demonstrated that the nucleates of oligomers do not seed fibril formation out of monomeric Aβs^52^. On the other hand, our simulations suggest that the collapsed state and extended state co-exist in Aβ conformation ensembles. It is thus more likely that nucleates of fibrils and oligomers originate from different monomeric conformations. An appropriate condition induces a particular form of nucleate that determines the consequent structure of fibril or oligomer. If further verified experimentally, the theory of Aβ assembly proposed here would provide valuable understanding and new manipulating methods to amyloid aggregation.

## Methods

### System setup

The coil-starting and helix-starting conformations were based on NMR structures of Aβ_10–35_ in water (pdb code: 1hz3)^24^ and that of Aβ_1–40_ in a water-micelle environment (pdb code: 1ba4)^29^. The first ten models of each PDB entry were used, upon which the rest of residues were added manually in PyMol to create the desired length. Each of the forty generated peptides was then put in centre of a cubic box with box edge 2.0 nm away from the peptide. Each cubic box was solvated with water molecules using simple point charge (SPC) model and neutralized by ions (3 Na^+^).

### MD simulation

The simulations of the forty Aβ systems were carried out with GROMACS 4.5 package^53^, applying GROMOS 53a6 force field on the peptides and solvents^54^. Periodic boundary conditions were used in x, y and z directions. The LINCS method^55^ was used to restrain all bonds, allowing a safe integration step of 2 fs. Electrostatic interactions were computed using the Particle Mesh Ewald (PME) method^56^. Lennard-Jones and Coulomb cut-off distances were set to 1.4 nm and 1.2 nm respectively. To energy-minimize the configurations, all simulations were subjected to the same four-step minimization with positional restrains imposed on (i) heavy atoms, (ii) main-chain atoms, (iii) Cα atoms and (iv) no atoms, step-wise. In the end, a maximum force less than 100 kJ.mol^−1^.nm^−1^ was obtained for each system. Four steps of thermalization in NPT ensemble, each lasting 500 ps, were subsequently implemented. The peptide and the solvent were coupled separately to a V-rescale thermostat^57^ at 300 K with positional restrains applied in the same order as that in the energy minimization. The pressure was kept constant at 1 bar by coupling to a berendsen barostat with τ_P _= 1.0 ps and a compressibility of 4.6 × 10^−5^ bar^58^. In the production runs, all parameters were set to the same as the last step of thermalization and the coordinates of the systems were saved every 100 ps. Table 1 lists the lengths of the simulations on Aβs. The simulations in negative control set were carried out for 500 ns.

### Back-calculation of J-coupling values and residual dipole constants

The Karplus equation was used to calculate the 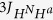 constant from our simulation coordinates^59^: 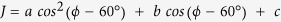, where *ϕ* is the peptide dihedral angle. The experimental J coupling data were obtained from Yan *et al.*^39^. The motional average effects were explicitly taken into account by fitting the Karplus coefficient to experimental data. Various published data sets were explored^35^,^60^,^61^,^62^, the calculated RMSD ranging from 0.7 Hz to 1.5 Hz. The best fitting was observed using parameters reported by Sgourakis *et al.*^35^, in which a, b and c are 7.7, −1.9 and 0.06 respectively.

The residual dipole constants from our simulations coordinates were calculated using PALES^40^. Calculation was implemented on snapshots every 1 ns and averaged within trajectories. The correlation between the experimental values obtained by Yan *et al.*^39^ and our back-calculated ones was calculated using Pearson correlation coefficient (P.C.C): 
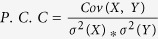
 , where *Cov*(*X,Y*) is the covariance of the two variables, and 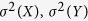 are the standard deviations.

### Spectral clustering

To find a small number of representative conformations for our large conformation ensembles, we employed a spectral clustering technique based on contact maps that was introduced by Sgourakis *et al.*^35^. We confirmed a contact when the COMs of two residues main-chains are closer than 4.5 Å, and denotes it as “1”; otherwise, a non-contact as “0”. The pair-wise contact information of one conformation then is expressed as a binary vector. The binary matrix that contains the contact information from one simulation group was transformed into square affinity matrix whose elements were the ‘distance’ between pairs of conformations. Singular value decomposition on the affinity matrix gave out high discriminative eigenvectors that can be used for data clustering and data visualization. We used the seven most discriminative eigenvectors for data clustering and the three most for visualization (Fig. S11).

The k-means/k-medoid method was used during data clustering and implemented by Pycluster module of Python. The within-cluster sum of distance was monitored as a function of cluster numbers. The number of clusters in each group was set after the turning point of the curve of cluster numbers vs. within-cluster sum distance (Fig. S11). Eventually we chose 10 clusters for each group.

## Additional Information

**How to cite this article**: Song, W. *et al.* Varied Probability of Staying Collapsed/Extended at the Conformational Equilibrium of Monomeric Aß_40_ and Aß_42_. *Sci. Rep.* **5**, 11024; doi: 10.1038/srep11024 (2015).

## Supplementary Material

Supplementary Information

## Figures and Tables

**Table 1:**
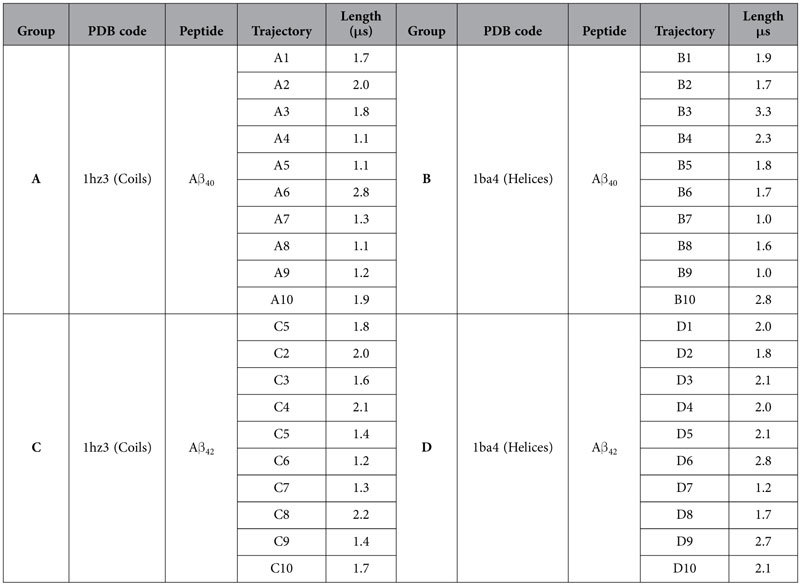
Configuration of the simulations.
