# Supplementary material for: Varied Probability of Staying Collapsed/Extended at the Conformational Equilibrium of Monomeric Aβ40 and Aβ42
Source: Sci Rep. 2015 Jun 5;5:11024. doi: 10.1038/srep11024 (PMC4603783; doi:10.1038/srep11024)
Supplement: Supplementary Information [file srep11024-s1.pdf]

Supplementary Information

Varied Probability of Staying Collapsed/Extended at the Conformational Equilibrium of Monomeric Aβ_{40} and Aβ_{42}

Wanling Song, Yuanyuan Wang, Jacques-Philippe Colletier, Huaiyu Yang, Yechun Xu

Corresponding email: ycxu@simm.ac.cn

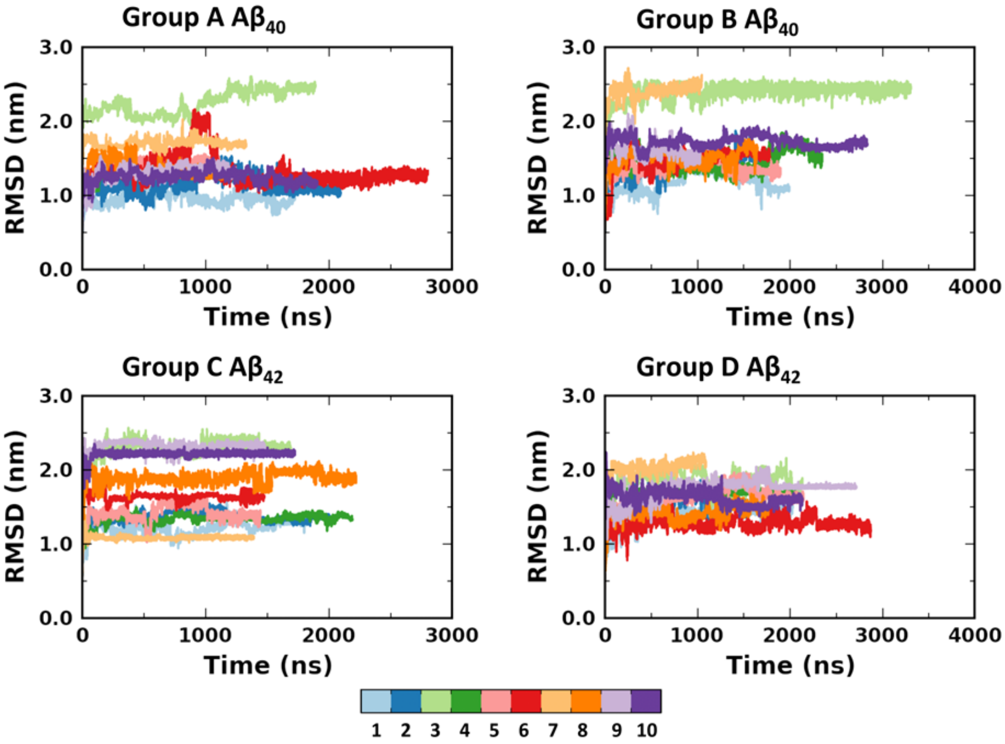

Figure S1. RMSD as a function of time. The ten simulations in each group are denoted by different colors.

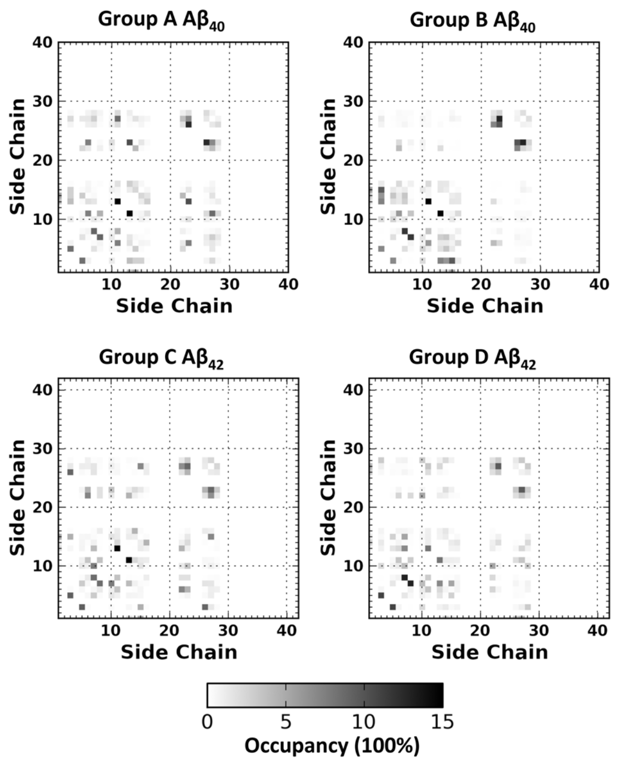

Figure S2. The occupancy of pair-wise side-chain hydrogen bonds in the four simulation groups.

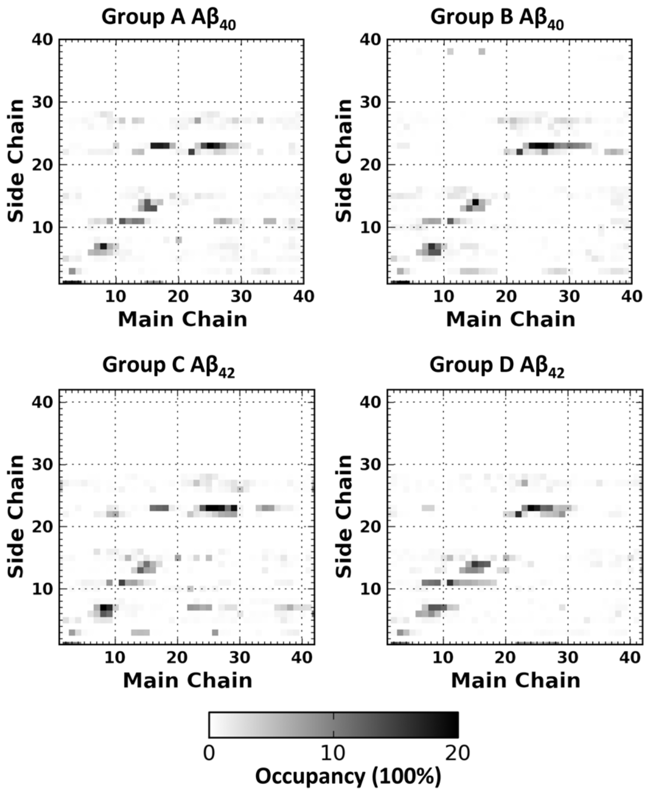

Figure S3. The occupancy of pair-wise hydrogen bonds between side-chain and main-chain in the four simulation groups.

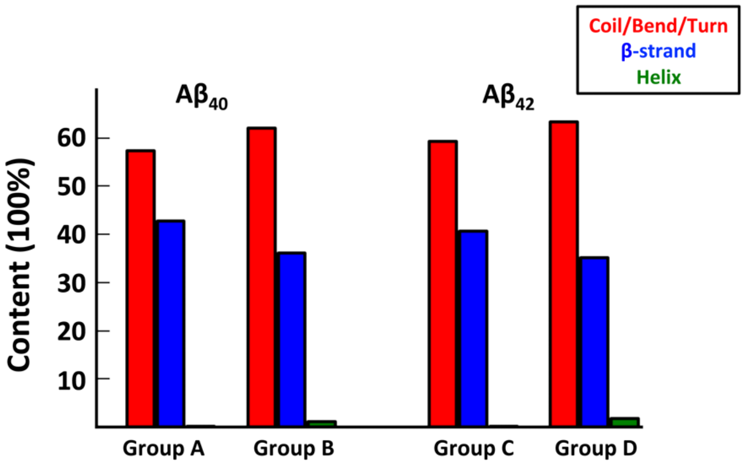

Figure S4. The content of secondary structure elements in the four simulation groups.

Table S1. Sencondary structure content.

	Content* (coil/β-strand/helix)	Standard Deviation** (coil/β-strand/helix)
Goup A (Aβ_{40})	0.572/0.426/0.002	0.207/0.207/0.002
Goup B (Aβ_{40})	0.619/0.369/0.011	0.186/0.185/0.014
Group C (Aβ_{42})	0.593/0.406/0.001	0.219/0.219/0.002
Group D (Aβ_{42})	0.632/0.351/0.016	0.195/0.195/0.023

* The values are the average among the ten simulations in each group.

** The values are the standard deviations among the ten simulations in each group.

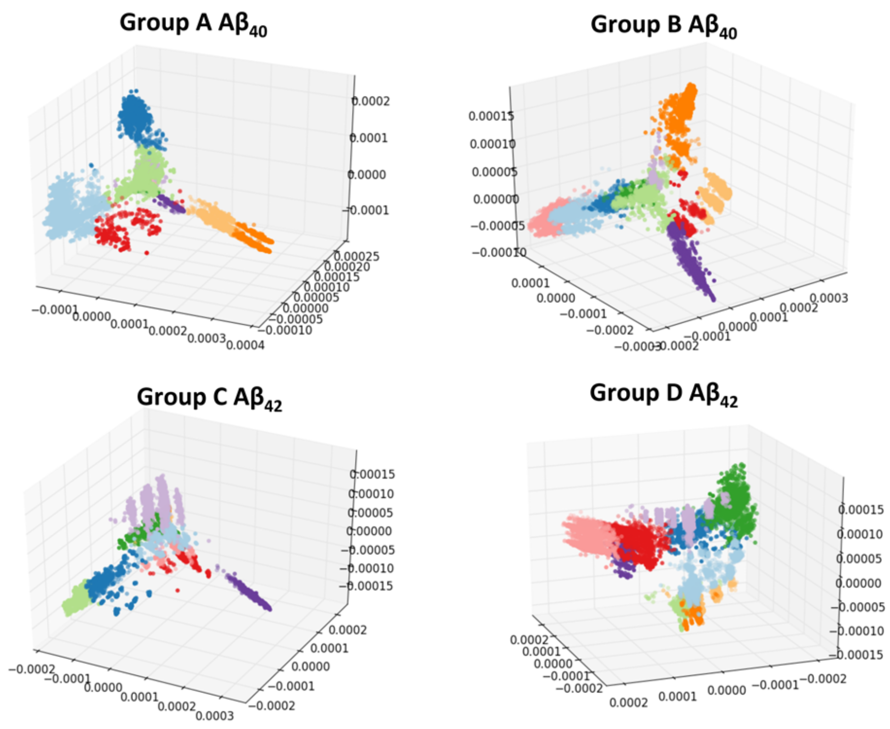

Figure S5. Visualization of the conformations in the 3D space defined by the three most discriminative eigenvectors. 10 clusters were generated for each data set and denoted by different colors.

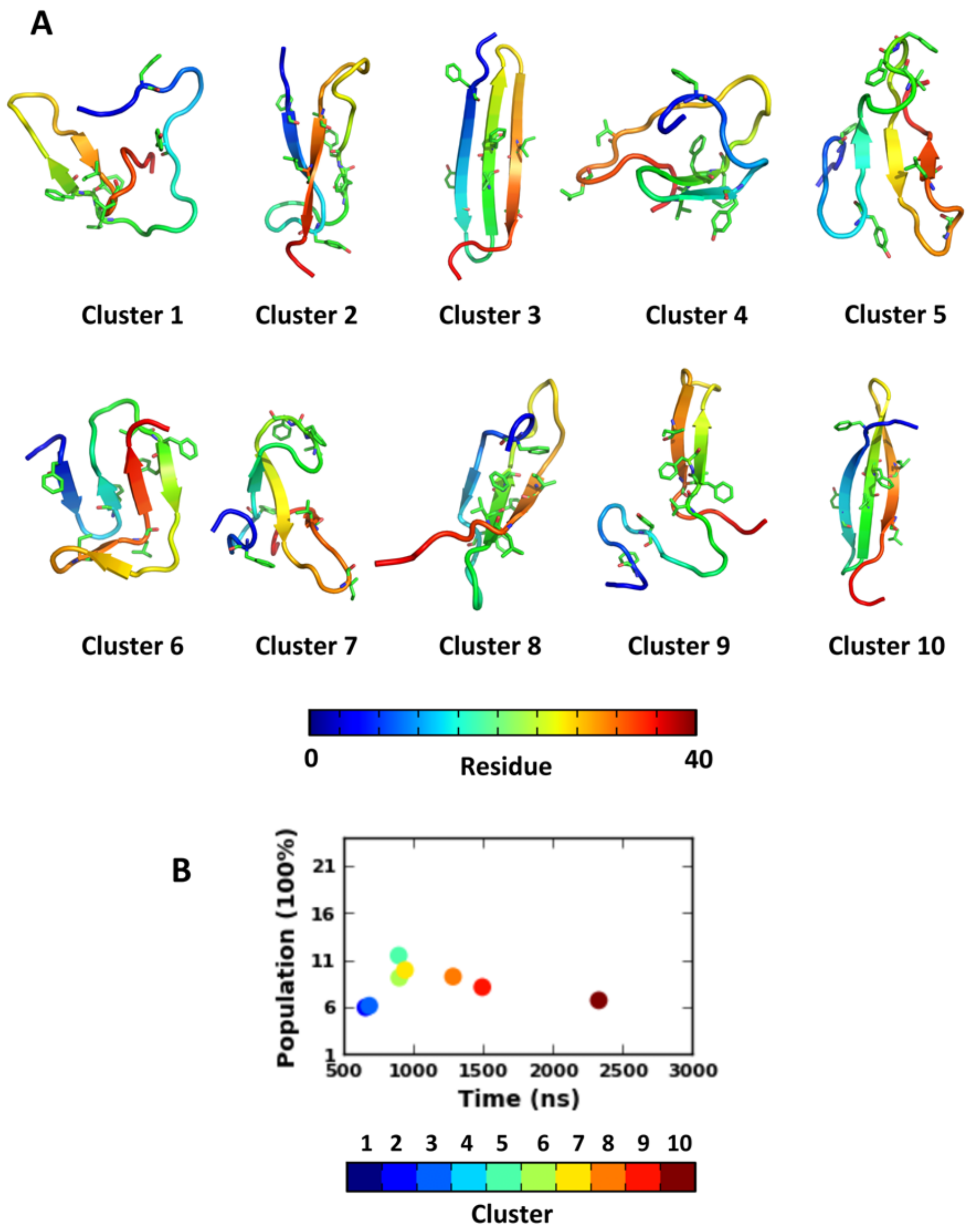

Figure S6. Spectral clustering of Aβ_{40} in Group A. A) The representative conformation of each clustered association pattern. The clusters are numbered sequentially based on their average appearance time in the course of simulations. B) The average appearance time of the clustered association patterns and the probability of finding each association pattern in conformation ensemble of Group A.

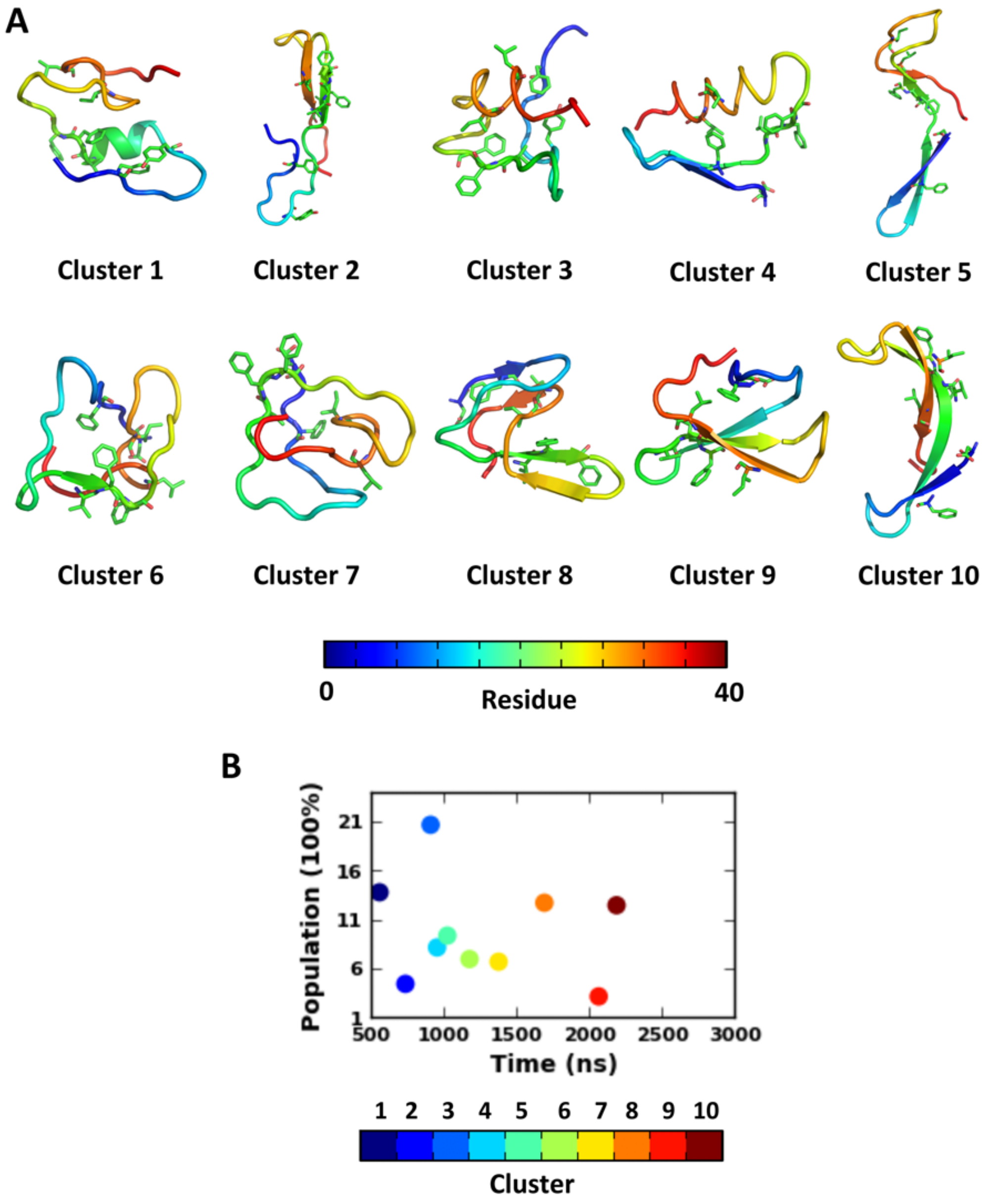

Figure S7. Spectral clustering of Aβ_{40} in Group B. A) The representative conformation of each clustered association pattern. The clusters are numbered sequentially based on their average appearance time in the course of simulations. B) The average appearance time of the clustered association patterns and the probability of finding each association pattern in conformation ensemble of Group B.

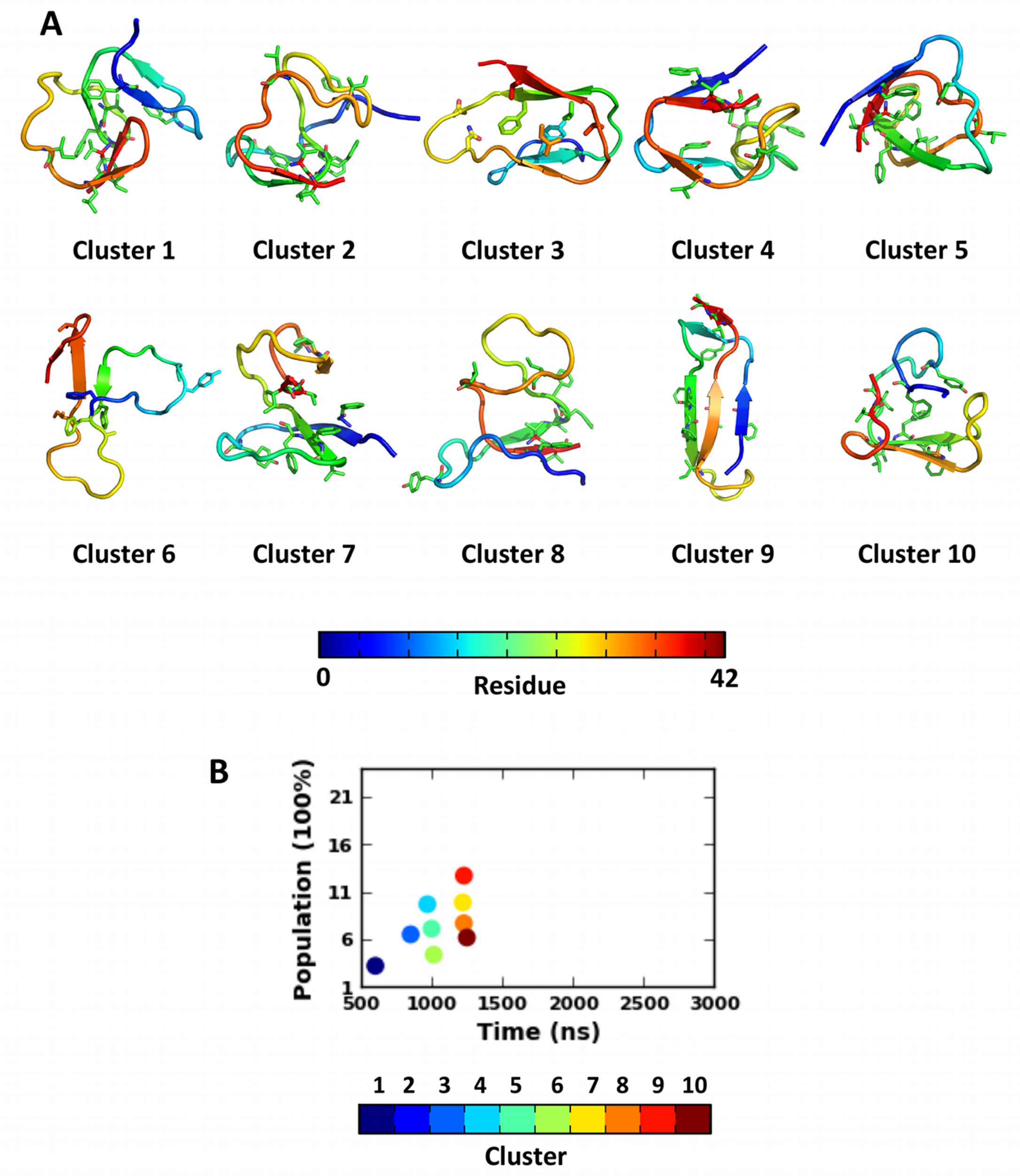

Figure S8. Spectral clustering of Aβ_{42} in Group C. A) The representative conformation of each clustered association pattern. The clusters are numbered sequentially based on their average appearance time in the course of simulations. B) The average appearance time of the clustered association patterns and the probability of finding each association pattern in conformation ensemble of Group C.

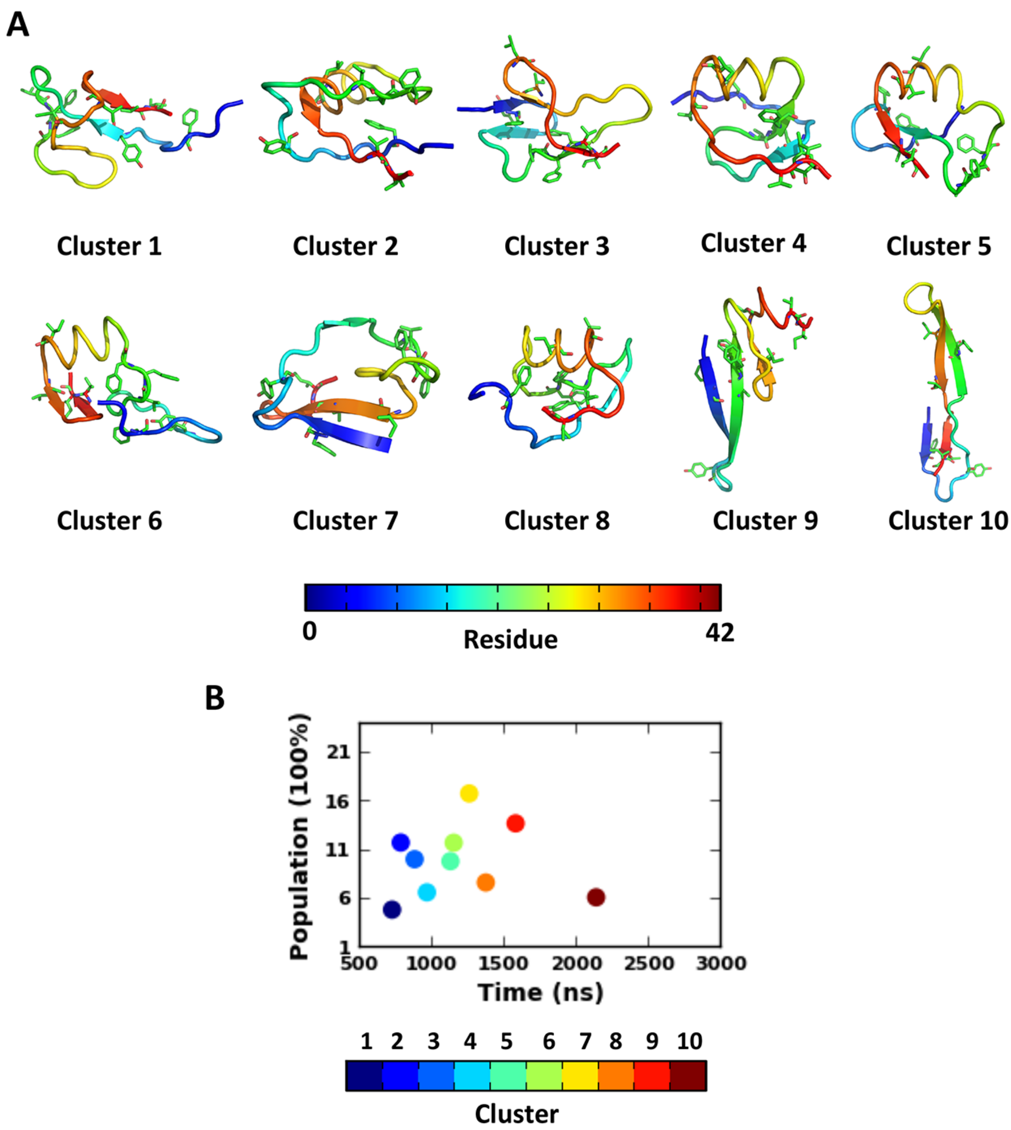

Figure S9. Spectral clustering of $A\beta_{42}$ in Group D. A) The representative conformation of each clustered association pattern. The clusters are numbered sequentially based on their average appearance time in the course of simulations. B) The average appearance time of the clustered association patterns and the probability of finding each association pattern in conformation ensemble of Group D.

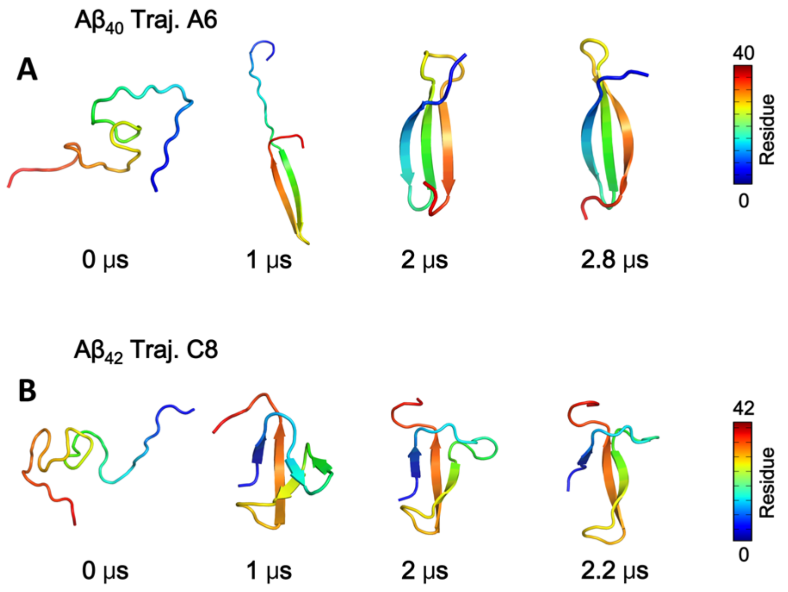

Figure S10. Snapshots of Traj. A6 (A) and Traj. C8. These two simulations have the larges content of β-strands in the simulations of $A\beta_{40}$ and $A\beta_{42}$, respectively.

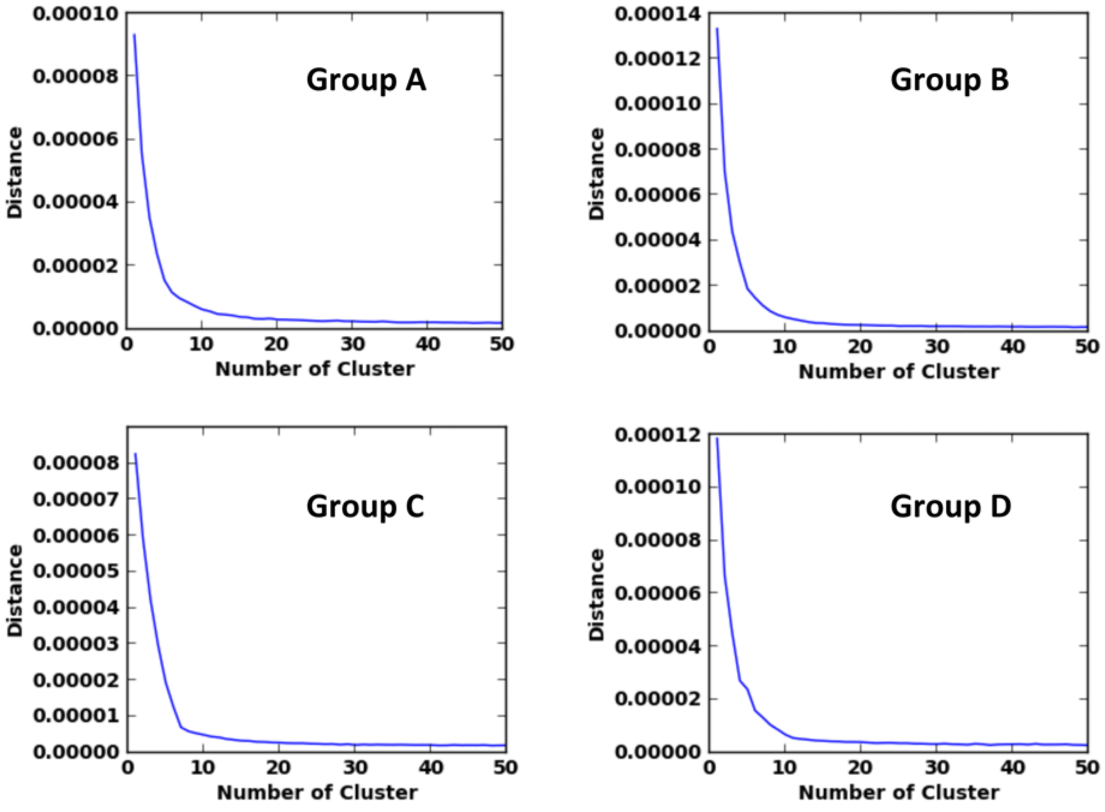

Figure S11. The within-distance sum of distance as a function of cluster numbers.
